# Changes in self‐reported symptoms of depression and physical well‐being in healthy individuals following a Taiji beginner course – Results of a randomized controlled trial

**DOI:** 10.1002/brb3.429

**Published:** 2016-03-04

**Authors:** Agnes Maria Schitter, Marko Nedeljkovic, Brigitte Ausfeld‐Hafter, Johannes Fleckenstein

**Affiliations:** ^1^ University of BernInstitute of Complementary Medicine IKOMBernSwitzerland; ^2^ Goethe‐University FrankfurtInstitute of Sports MedicineFrankfurtGermany

**Keywords:** Mind‐body, mindfulness, mood, quality of life, RCT, traditional Chinese medicine

## Abstract

**Background:**

Taiji is a mind–body practice being increasingly investigated for its therapeutic benefits in a broad range of mental and physical conditions. The aim of this study was to investigate the potential preventive effects of Taiji practice in healthy individuals with regard to their depressive symptomatology and physical well‐being.

**Methods:**

Seventy healthy Taiji novices were randomly assigned to a Taiji intervention group, that is, Taiji beginner course (Yang‐Style Taiji, 2 h per week, 12 weeks) or a control group comprised of the waiting list for the course. Self‐reported symptoms of depression (CES‐D) and physical well‐being (FEW‐16) were assessed at baseline, at the end of the intervention, as well as 2 months later.

**Results:**

The included participants had a mean age of 35.5 years. Physical well‐being in the Taiji group significantly increased when comparing baseline to follow‐up (FEW‐16 sum score *T*(27) = 3.94, *P = *0.001, 95% CI 0.17 to 0.55). Pearson's correlation coefficients displayed a strong negative relationship between self‐reported symptoms of depression and physical well‐being (*P*'s < 0.001, *r*'s ≥ −0.54).

**Conclusion:**

In this randomized controlled trial, we found significant evidence that a Taiji beginner course of 3 months duration elicits positive effects with respect to physical well‐being in healthy individuals, with improvements pronouncing over time. Physical well‐being was shown to have a strong relationship with depressive symptoms. Based on these results, the consideration of Taiji as one therapeutic option in the development of multimodal approaches in the prevention of depression seems justifiable.

## Introduction

### Background

Taiji – variably spelled Taijiquan, Tai Chi or Tai Chi Chuan – is one treatment modality of traditional Chinese medicine (TCM) having its roots in ancient Chinese martial arts (Frantzis 1997). From a western point of view, Taiji is regarded as a form of a mind–body practice (National Institutes of Health, 2008). Nowadays, Taiji is predominantly practiced as series of slow, graceful movements in combination with visualizations of intentional flow of the “qi” (life energy, vitality [Wayne and Kaptchuk 2008]). Suitable for trainees in a wide range of ages and levels of physical fitness, Taiji became increasingly popular in western countries in recent years not only as a physical activity, but also as a means of therapy (Barnes et al. 2004; Birdee et al. 2009). In reviews, various psychological benefits of Taiji were reported, for example, improvement of mental disorders (Abbott and Lavretsky 2013; Kim et al. 2013) improvement of mood, and reduction of anxiety, symptoms of depression, anger‐tension and perceived stress (Jimenez et al. 2012). Taiji is also attributed a variety of physical health benefits, especially in chronic conditions (Lan et al. 2013; Lauche et al. 2013). Traditionally, Taiji is ascribed preventive qualities as sustained fitness when practiced long term (Frantzis 1997). As a preventive modality, Taiji is mentioned in the scientific literature in conjunction with reduction in risk of falls in elderly persons (Gillespie et al. 2012) and fortification of the immune system (Ho et al. 2013).

Self‐reported well‐being is increasingly used as an indicator for health (Kolip and Schmidt 1999) and linked to health protection (Steptoe et al. 2014). It seems to be specifically impaired in depressed individuals (Hays et al. 1995). Since a reduction in symptoms of depression following Taiji practice was observed not only in individuals suffering major depression (Rosenbaum et al. 2014) but also in healthy populations (Brown et al. 1995; Dechamps et al. 2009) we investigated, whether the latter could be reproduced in our sample of healthy individuals. We also assessed whether taking up Taiji as a physical practice routine had an influence on physical well‐being and to what extent symptoms of depression are correlated with physical well‐being.

## Methods

### Study design

This study's data were collected in the course of a randomized controlled trial investigating psychobiological stress reactivity (Nedeljkovic et al. 2012). Ethical approval was given by the Ethics‐Committee of the Canton Bern, Switzerland. Healthy participants between 18 and 50 years of age were recruited by advertisement at pin‐boards of the University of Bern from April 2010 to August 2010. They underwent a telephone screening controlling for the following exclusion criteria: somatic or mental disorders, intake of medication or addictive substances, previous Taiji experience, and predictable absence of more than 1 week during the intervention period. Eligible participants who handed in written consent based on oral and written information were included in the study and randomly assigned to the intervention group or the control group. Allocation concealment was achieved by using sequentially numbered, opaque and sealed envelopes.

Psychometric data were collected using online questionnaires in a baseline assessment, right at the end of the 12 week intervention period, and at a follow‐up assessment 2 months after the end of the intervention (week 20).

### Taiji intervention

Participants in the intervention group attended a Taiji beginner course over a period of 12 weeks, attending two 60 min course lessons per week and being encouraged to regularly practice Taiji at home. Course attendance was monitored by the teacher.

In this Taiji beginner course, study participants were taught consecutively the first 18 motion sequences of the 37 Zheng Manqing Yang‐Style Taiji short form (Robinson 2009). These sequences are suited to convey the fundamental principles of Taiji such as effortless alignment of the body and holistic awareness during physical movement (Wolf et al. 1997).

All course lessons were led by a certified Taiji teacher (MN), licensed by the Swiss Society for Qigong and Taijiquan [Schweizerische Gesellschaft für Qigong und Taijiquan – SGQT]. Lessons started with 15 min of warm up, followed by 35 min of commented Taiji practice and 10 min of Taiji‐related breathing and relaxation exercises.

All participants were requested to refrain from any additional new physical exercise or mind–body program during their study participation. After completion of the randomized controlled trial, an equivalent Taiji course was offered to the control group.

### Outcomes

Sociodemographic parameters, that is, gender, age, BMI, smoking as well as regular physical activity and participants' prior experience in mind–body practice (e.g., meditation, yoga, etc.) were assessed at baseline.

Depression and negative thought patterns were assessed using the German version of the ‘‘Center for Epidemiological Studies Depression Scale’’ (CES‐D), the ADS‐K [Allgemeine Depressions‐Skala] questionnaire (Radloff 1977; Hautzinger and Bailer 1993). This 15 item questionnaire was developed for research in the general population depicting affective, motivational, and cognitive aspects of depression. Its scores range between 0 and 45, with higher values indicating increased symptoms of depression. The internal consistency of this instrument was reported to be very good (Cronbach's α  =  0.95) (Lehr et al. 2008).

Physical well‐being was assessed by means of the “Questionnaire for Assessing Subjective Physical Wellbeing” FEW‐16 [Fragebogen zur Erfassung körperlichen Wohlbefindens] (Kolip and Schmidt 1999). By measuring well‐being rather than the absence of distress or deficits, this questionnaire adopts a salutogenetic approach and comprises four subscales: stress resistance, vitality, ability to enjoy, and inner peace. Its 16 positively worded items cover values ranging from 0 to 5, with 0 referring to lowest and 5 referring to highest agreement. The averaged total score ranges from 0 to 5, with higher values indicating higher perceived physical well‐being. Reported internal consistency values are Cronbach's α = 0.92 for the total scale and 0.82 to 0.90 for the subscales (Kolip and Schmidt 1999).

### Statistical analyses

Data analysis was conducted using the statistical software package SPSS version 21 for Microsoft (IBM SPSS Statistics, Somers, NY). Employing G*Power 3.1 (Buchner et al. 1997), the total sample size of *n* = 68 (with power ≥ 0.80 and α = 0.05) was estimated a priori to detect a medium to large effect as reported in Taiji studies on depression (Wang et al. 2013; Zeng et al. 2014). All analyses were two‐tailed, with the level of significance set at *P *<* *0.05 with a confidence interval CI of 95%. Unless indicated, all results are presented as mean ± standard deviations (SD). Effect size parameters (*f*) were calculated from partial *η* ^2^ values where appropriate, and reported in accordance to effect size convention (Cohen 1988), that is, *f*: 0.10 = small, 0.25 = medium, 0.40 = large.

Prior to statistical analyses, all data were tested for homogeneity of variance and normal distribution employing the Levene and Kolmogorov–Smirnov test. Group characteristics were analyzed using independent samples *t*‐test for continuous data, and χ^2^‐test for categorical data. Group differences in baseline values were also tested employing *t*‐tests. Data were analyzed per protocol up to the end of the intervention period. For missing follow‐up values, the last value was carried forward. The statistician conducting the analyses was blinded.

Paired sample *t*‐tests were applied to examine the impact of Taiji on symptoms of depression (ADS‐K) and on physical well‐being (FEW‐16) from baseline to postintervention and from baseline to follow‐up. As baseline values significantly differed between the two study groups, results were confirmed by one‐way ANCOVAs with group (Taiji, control) as independent variable and mean change values (from baseline to postintervention and from baseline to follow‐up) in ADS‐K and FEW‐16 scores as dependent variables, with respective baseline values serving as covariates. To test whether course participation frequencies had an influence on the achieved results, we conducted exploratory analyses by calculating Pearson's correlation coefficients between course participation frequency and mean change values of ADS‐K or FEW‐16 for both the interval pre‐ to postintervention and preintervention to follow‐up.

To explore the relationship between symptom severity of depression and the degree of physical well‐being, Pearson's correlation coefficients were calculated between the sum scales of ADS‐K and FEW‐16 and its four subscales on the three time points of data collection, that is, baseline, post, and follow‐up.

## Results

### Process of study participation and adherence to the intervention

Out of 112 applicants interested in study participation, 42 subjects did not meet inclusion criteria and thus where not included. For further details, please see Nedeljkovic et al. (2012). The remaining 70 eligible subjects were enrolled and randomly assigned to either the Taiji group (*n* = 35) or the control group (*n* = 35). In the Taiji group, seven participants dropped out during the intervention period and one was lost to follow‐up. In the control group, four discontinued prior to postintervention assessment and one was lost to follow‐up. A detailed description of dropout reasons and a CONSORT flow chart are presented elsewhere (Nedeljkovic et al. 2012). Eventually, data from 28 participants in the Taiji group and 31 participants in the control group were included in the final analyses. Participants in the Taiji group attended 20.8 ± 2.7 (86.8%, range 15–24) of the 24 lessons given.

### Baseline characteristics

Participants under study were healthy, well‐educated adults with >75% holding a high school degree. The two study groups did not differ significantly in terms of age (mean age 35.5 years), gender (one‐third of the participants were male), body mass index, education, occupational status, or smoking habits (Nedeljkovic et al. 2012). As shown in Table 1, they were also comparable with regard to the presence of symptoms of depression as represented by the mean values of the ADS‐K sum score. Participants in the Taiji group reported significantly higher physical well‐being at baseline (*P = *0.030), scoring particularly higher on the FEW‐16 subscale stress resistance (*P = *0.025).

**Table 1:** Mean values at baseline, postintervention, and follow‐up

Mean values^a^	Taiji group (*n* = 28)			Control group (*n* = 31)			*P* b
	Baseline	Postintervention	Follow‐up	Baseline	Postintervention	Follow‐up	
ADS‐K sum score	10.46 ± 6.90	8.68 ± 7.81	8.25 ± 8.47	10.74 ± 7.10	11.74 ± 7.05	8.68 ± 7.31	0.880
FEW‐16 sum score	3.22 ± 0.55	3.39 ± 0.63	3.58 ± 0.63	2.88 ± 0.63	2.98 ± 0.73	3.03 ± 0.76	0.030
FEW‐16 subscale stress resistance	3.85 ± 0.73	3.88 ± 0.58	4.04 ± 0.57	3.40 ± 0.78	3.44 ± 0.88	3.36 ± 1.01	0.025
FEW‐16 subscale ability to enjoy	3.40 ± 0.94	3.63 ± 0.79	3.83 ± 0.80	3.19 ± 0.71	3.37 ± 0.74	3.44 ± 0.66	0.339
FEW‐16 subscale vitality	2.93 ± 0.93	3.13 ± 1.07	3.20 ± 0.94	2.51 ± 0.97	2.60 ± 1.20	2.60 ± 1.31	0.095
FEW‐16 subscale inner peace	2.71 ± 0.84	2.91 ± 0.93	3.26 ± 1.01	2.41 ± 0.93	2.52 ± 1.09	2.72 ± 1.08	0.209

a Data are expressed as mean ± SD.

b *P*‐values refer to independent samples *t*‐tests for baseline characteristics.

ADS‐K, German version of CES‐D questionnaire; FEW‐16, Questionnaire for Assessing Subjective Physical Well‐being. High values indicate pronounced symptoms of depression or physical well‐being, respectively.

**Table 2:** Development of self‐attributed symptoms of depression and physical well‐being between Taiji group and control group

	Taiji group (*n* = 28)	Control group (*n* = 31)	*P* b	Partial Eta^2^
Δ values^a^ from pre‐ to postintervention				
ADS‐K sum score	−1.79 ± 4.97	1.00 ± 6.25	0.047	0.068
FEW‐16 sum score	0.17 ± 0.44	0.11 ± 0.51	0.38	0.014
FEW‐16 subscale stress resistance	0.04 ± 0.59	0.05 ± 0.66	0.36	0.015
FEW‐16 subscale ability to enjoy	0.22 ± 0.60	0.18 ± 0.70	0.40	0.012
FEW‐16 subscale vitality	0.21 ± 0.82	0.10 ± 0.93	0.40	0.013
FEW‐16 subscale inner peace	0.21 ± 0.82	0.11 ± 0.74	0.41	0.012
Δ values^a^ from baseline to follow‐up				
ADS‐K sum score	−2.21 ± 6.72	−2.06 ± 6.61	0.89	0.000
FEW‐16 sum score	0.36 ± 0.49	0.15 ± 0.55	0.058	0.062
FEW‐16 subscale stress resistance	0.20 ± 0.59	−0.03 ± 0.83	0.039	0.074
FEW‐16 subscale ability to enjoy	0.43 ± 0.78	0.24 ± 0.53	0.065	0.059
FEW‐16 subscale vitality	0.27 ± 0.76	0.10 ± 1.14	0.25	0.024
FEW‐16 subscale inner peace	0.55 ± 0.85	0.31 ± 0.72	0.14	0.038

a Continuous data are expressed as mean ± SD.

b *P*‐values refer to between‐group effects in one‐way ANCOVAs.

ADS‐K, German version of CES‐D questionnaire; FEW‐16, Questionnaire for Assessing Subjective Physical Well‐being. Positive change values indicate increased symptoms of depression or physical well‐being, respectively.

### Changes in self‐reported symptoms of depression and physical well‐being

#### Symptoms of depression

Within‐group analyses showed a trend toward a decrease in symptoms of depression in the Taiji group from baseline to postintervention (*T*(27) = −1.90, *P = *0.068, 95% CI = −3.71 to 0.14), which persisted at follow‐up assessment (*T*(27) = −1.74, *P = *0.093, 95% CI = −4.82 to 0.39). Participants in the control group reported nonsignificant changes in ADS‐K values from baseline to postintervention and a trend toward a decrease in depressive symptomatology at follow‐up assessment.

Since the confidence intervals cross zero, effects on symptoms of depression as assessed with ADS‐K scores remain inconclusive.

#### Physical well‐being

**Figure 1:**
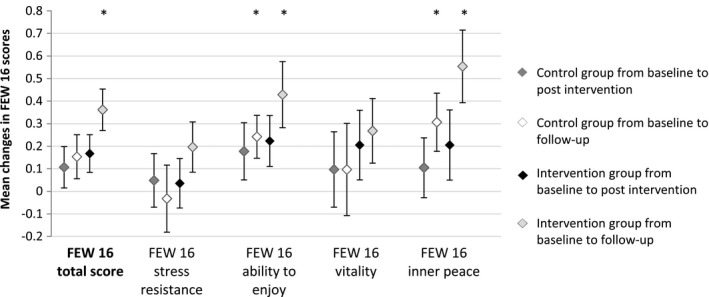
Development of self‐attributed physical well‐being: FEW‐16 in Taiji group and control group. Values are means ± standard error of the mean (SEM) and refer to paired sample *t*‐tests. Positive values indicate increased well‐being. Significance is indicated by an asterisk (*P* < 0.05). *FEW‐16*: Questionnaire for Assessing Subjective Physical Well‐being.

Within the Taiji group (see Fig. 1), a trend toward an increase in self‐reported physical well‐being from baseline to postintervention was observed, which became significant at the follow‐up assessment (*T*(27) = 3.94, *P = *0.001, 95% CI 0.17 to 0.55). This increase in the FEW‐16 sum score from baseline to follow‐up was mainly related to the improvements in the FEW‐16 subscales “ability to enjoy” (*T*(27) = 2.93, *P = *0.007, 95% CI 0.13 to 0.73) and “inner peace” (*T*(27) = 3.44, *P = *0.002, 95% CI 0.22 to 0.88). With respect to the control group, no significant changes in FEW‐16 sum scores were found in the course of the study (*P *>* *0.05). However, from baseline to follow‐up, participants in the control group reported a significant increase in the FEW‐16 subscales “ability to enjoy” (*T *=* *2.54, *P = *0.016, 95% CI 0.05 to 0.44) and “inner peace” (*T *=* *2.38, *P = *0.024, 95% CI 0.04 to 0.57).

In comparison to the control group (see table 2) a trend toward a higher mean change value of the FEW‐16 sum score was found in the Taiji group from baseline to follow‐up (*F*(1/56) = 3.73; *P = *0.058; *f *=* *0.26). In particular, the group differences in the mean change value of the FEW‐16 subscales “stress resistance” (*F*(1/56) = 4.46; *P = *0.039; *f *=* *0.28) and “ability to enjoy” (*F*(1/56) = 3.53; *P = *0.065; *f *=* *0.25) contributed to this trend.

Course participation frequency did not correlate significantly with ADS‐K or FEW‐16 mean change values (*p *>* *.147).

### Correlations between self‐reported symptoms of depression and physical well‐being

Exploratory analyses revealed strong significant negative relationships between ADS‐K and FEW‐16 sum scores at all points of measurement (*P*'s < 0.001): baseline, postintervention and at follow‐up (*r = −*0.54, *r = −*0.64, and *r = −*0.64, respectively). ADS‐K sum score correlated significantly with three out of the four FEW‐16 subscale scores at all points of measurement (*P*'s < 0.05): inner peace (pre: *r = −*0.59, post: *r = −*0.65, follow‐up: *r = −*0.69), ability to enjoy (pre: *r = −*0.41, post: *r = −*0.52, follow‐up: *r = −*0.51), and vitality (pre: *r = −*0.31, post: *r = −*0.54, follow‐up: *r = −*0.45). FEW‐16 subscale stress resistance values correlated significantly with the ADS‐K sum score at follow‐up (*r = −*0.31, *P = *0.017), but not at baseline (*r = −*0.20, *P = *0.136) and postintervention (*r = −*0.15, *P = *0.273).

## Discussion

This randomized controlled trial examines the impact of Taiji on self‐attributed symptoms of depression and physical well‐being in healthy individuals. Novices in a Taiji beginner course were monitored during an intervention period of 3 months and a follow‐up period of 2 months.

Rooted in martial arts, Taiji comprises a multitude of movements and positions that can be regarded as “power‐postures” that have the potential to elicit changes in the neuroendocrinological system within minutes (Carney et al. 2010). Accordingly, modified hormonal activities along with changes in behavior and attitude toward increased awareness and self‐compassion were reported about participants in the currently described trial (Nedeljkovic et al. 2012; Schitter et al. 2013), emphasizing the increase in effects regarding self‐compassion beyond the designated training period. Behavior and lifestyle are being discussed not only with regard to treatment of depression, but also to its onset (Sarris et al. 2014). While reduction in symptoms of depression following Taiji practice has been reported (Wang et al. 2010, 2013; Zeng et al. 2014), Taiji as a means to depression prevention – a clinically and health economically highly relevant issue – remains a rarely addressed subject.

Following traditional perception (Frantzis 1997) as well as scientific observation (Chen et al. 2002; Gyllensten et al. 2010), participants in the Taiji beginner training in our trial had been explicitly encouraged and instructed to practice independently beyond the guided classes and to develop a practice routine that they might maintain once this supportive setting would no longer be available to them. More pronounced effects under long‐term practice might be a reason, why participants displayed a nonsignificant trend (*P = *0.058) of experiencing larger positive changes on the FEW‐16 sum scale (self‐attributed physical well‐being) from baseline to follow‐up than the control group.

Based on the literature discussing the influence of depression on physical well‐being (Hays et al. 1995), we expected a negative correlation between ADS‐K and FEW‐16. In fact, we found significant large correlations between ADS‐K and FEW‐16 sum scores, as well as between ADS‐K score and the FEW‐16 subscales inner peace, ability to enjoy, and vitality.

The following limitations need to be addressed. First, despite randomization, baseline differences between the two study groups were present in the FEW‐16 sum score and in its subscale stress resistance. We accounted for this matter in statistical analyses.

Secondly, the design of the trial did not consider control for additional social contacts that inevitably come along with the training in a group and might have influenced the results. Since this trial did focus on efficacy research, no active control group was involved. Future trials on comparative effectiveness should have an exercise control group, which will solve this problem.

The main strengths of this study are its randomized controlled design with a follow‐up period of 2 months and the application of restrictive exclusion criteria, both contributing to a high internal validity.

## Conclusion

In this randomized controlled trial, we found significant evidence that a Taiji beginner course of 3 months duration elicits positive effects with respect to physical well‐being in healthy individuals. The overall negative correlation between self‐reported symptoms of depression and physical well‐being was strong. Still, it is to determine to which extent long‐term use may further enhance these observations and how many training units per week would lead to optimal outcomes. Our results support the consideration to implement Taiji as one therapeutic option in the development of multimodal approaches in the prevention and therapy of depression.

## Conflict of Interests

All authors declare that they have no conflict of interest.
